# Role of endothelial permeability hotspots and endothelial mitosis in determining age-related patterns of macromolecule uptake by the rabbit aortic wall near branch points

**DOI:** 10.1016/j.atherosclerosis.2016.05.017

**Published:** 2016-07

**Authors:** K. Yean Chooi, Andrew Comerford, Stephanie J. Cremers, Peter D. Weinberg

**Affiliations:** a Department of Bioengineering, Imperial College London, London, SW7 2AZ, UK; b Department of Aeronautics, Imperial College London, London, SW7 2AZ, UK

**Keywords:** Atherosclerosis, Permeability, Transport, Albumin, Evans Blue dye, Aorta, Branch, Age

## Abstract

**Background and aims:**

Transport of macromolecules between plasma and the arterial wall plays a key role in atherogenesis. Scattered hotspots of elevated endothelial permeability to macromolecules occur in the aorta; a fraction of them are associated with dividing cells. Hotspots occur particularly frequently downstream of branch points, where lesions develop in young rabbits and children. However, the pattern of lesions varies with age, and can be explained by similar variation in the pattern of macromolecule uptake. We investigated whether patterns of hotspots and mitosis also change with age.

**Methods:**

Evans’ Blue dye-labeled albumin was injected intravenously into immature or mature rabbits and its subsequent distribution in the aortic wall around intercostal branch ostia examined by confocal microscopy and automated image analysis. Mitosis was detected by immunofluorescence after adding 5-bromo-2-deoxiuridine to drinking water.

**Results:**

Hotspots were most frequent downstream of branches in immature rabbits, but a novel distribution was observed in mature rabbits. Neither pattern was explained by mitosis. Hotspot uptake correlated spatially with the much greater non-hotspot uptake (*p* < 0.05), and the same pattern was seen when only the largest hotspots were considered.

**Conclusions:**

The pattern of hotspots changes with age. The data are consistent with there being a continuum of local permeabilities rather than two distinct mechanisms. The distribution of the dye, which binds to elastin and collagen, was similar to that of non-binding tracers and to lesions apart from a paucity at the lateral margins of branches that can be explained by lower levels of fibrous proteins in those regions.

## Highlights

• The pattern of permeability hotspots around aortic branch points changed with age.
• Non-hotspot, hotspot and large hotspot uptake all showed the same patterns.
• This is consistent with a continuum of permeabilities rather than two mechanisms.
• The patterns of hotspots were not explained by patterns of mitosis.
• The distribution of fibrous proteins influences patterns of Evans’ Blue in the wall.

## 1 Introduction

Elevated uptake of circulating macromolecules, particularly low density lipoprotein (LDL), by the arterial wall is seen in anatomical locations that are particularly susceptible to atherosclerosis and is thought to be a risk factor for it. The first study to show this used the intravital dye Trypan Blue [1], an isomer of Evans’ Blue dye (EBD), which binds chiefly to serum albumin in the circulation [2]; the dye was preferentially taken up by the flow divider at arterial branch points, a site that was already known to be particularly prone to lesions in the cholesterol-fed rabbit [3]. Although the study was conducted using frogs, the same result was subsequently obtained in young pigs [4].

The endothelium presents a substantial resistance to macromolecule transport into the wall. Possible routes across it include vesicular pathways and intercellular junctions. Normal junctions are ≤20 nm in width so although they should allow the passage of albumin (4 × 14 nm; [5]), LDL (Stokes-Einstein diameter 23 nm; [6]) is unlikely to enter the arterial intima via this route. The cell turnover leaky junction hypothesis of Weinbaum et al. [7] proposes that intercellular junctions temporarily widen when endothelial cells divide or die, leading to foci of high permeability for macromolecules such as LDL. Typical dimensions of these leaky junctions were estimated by Chen et al. [8] to be 80–1330 nm during mitosis and 15–1000 nm for dead or dying cells. The hypothesis is consistent with earlier observations that mitosis rates are higher in areas of Evans’ Blue dye-albumin (EBA) uptake by the pig aortic wall [9].

Stemerman et al. [10] observed that uptake of horseradish peroxidase (HRP, Stokes-Einstein diameter 6.4 nm; [11]) by the rabbit aorta occurred in distinct spots (here termed “hotspots”) 1 min after administration. Concentrations of LDL in the HRP hotspots were up to 47 times greater than in HRP-free arterial tissue, suggesting that LDL had crossed the endothelium via routes also taken by the much smaller HRP. Subsequently, Lin et al. showed that 99% of mitotic cells [12] and 63% of dead or dying cells [13] were associated with EBA hotspots. Furthermore, these mitotic and dead or dying cells accounted for 30% and 37%, respectively, of all EBA hotspots. (EBA is particularly suitable for hotspot studies because the EBD binds preferentially to elastin and collagen on entering the wall [14], thus leaving a permanent record of its site of entry rather than dispersing in the underlying tissue.) A study using ^125^I-LDL reported that 80% of mitotic cells were associated with LDL hotspots, and that mitotic cells were present in 45% of hotspots [15]. However, a similar study by Truskey et al. [16] found a weaker association, approximately 25% of leakage sites being associated with mitosis, and a further study from the same group [17] found that only 8% of LDL hotspots were associated with endothelial cells in S phase. Hotspots occur at a particularly high frequency downstream of branch points [18], [19].

It has emerged that the pattern of lesions in cholesterol-fed rabbits changes with age: although lesions occur downstream of aortic side branches in young animals, they occur more frequently at the sides and upstream of branches at later ages [20], [21]. A similar switch is seen in the spontaneous lesions that occasionally affect rabbit aortas [22] and it parallels a comparable change with age in human aortas [23], [24], [25]. Furthermore, the pattern of macromolecule uptake by the rabbit aortic wall also changes with age, and in the same way [26], [27], [28], [29], [30]. This concordance is important because it resolves inconsistencies between patterns of lesions and uptake that were apparent between earlier studies and provides additional evidence for a key role of transport properties in atherogenesis [31]. However, the effect of age on the pattern of hotspots and its dependence on mitosis has not been examined.

The present study investigated the hypothesis that patterns of EBA hotspots and mitosis change with age in rabbits in the same way as lesions, and examined the proportion of total uptake that occurs via hotspots. The study employed a range of technical innovations: EBD was detected from its fluorescence rather than its absorbance in order to increase sensitivity; *en face* confocal microscopy was used in conjunction with a maximum intensity projection in order to preferentially detect EBD bound to elastin in the inner wall; hotspots were identified and their area and intensity were quantified by an objective, automated method of image segmentation; mitosis occurring over several days rather than solely at the time of death was identified by adding a DNA synthesis marker to drinking water; and comparisons between patterns of hotspots, mitosis and lesions were made by rigorous statistical methods [32] that account for autocorrelation and avoid assumptions of linearity.

## 2 Materials and methods

Methods and their validation are given in the on-line Supplementary data. All animal procedures complied with the Animals (Scientific Procedures) Act 1986 and were approved by the Local Ethical Review Panel of the University of Reading.

## 3 Results

### 3.1 Hotspots

**Fig. 1:**
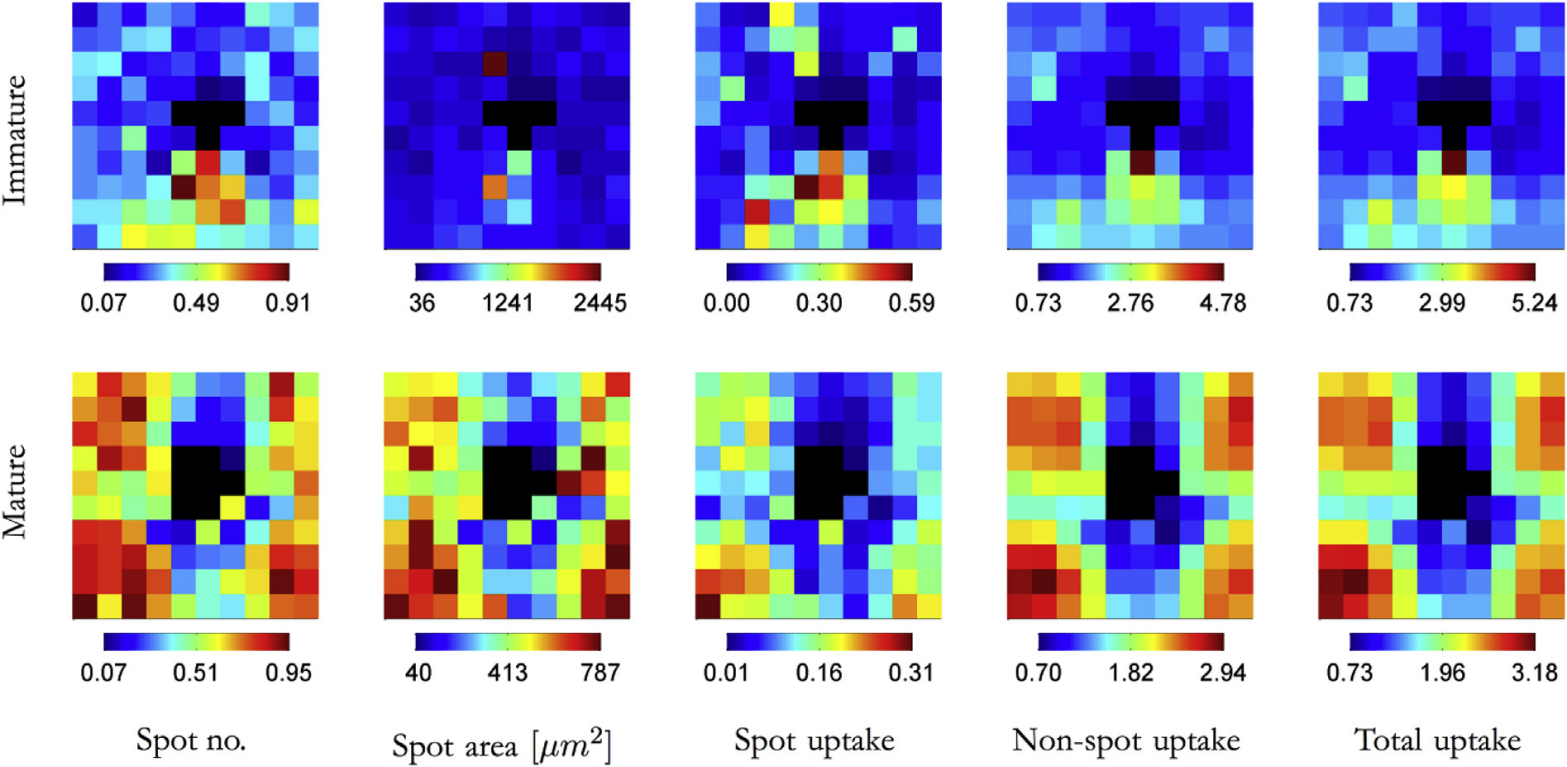
Maps of EBA uptake. Average EBA uptake maps for 63 branches from 5 immature rabbits and 58 branches from 5 mature rabbits. The maps show a 2.4 × 2.4 mm area of the aortic wall, displayed en face and centred on the intercostal ostium, with mean aortic blood flow from top to bottom. Each map is divided into 10 × 10 grid squares. The black squares at the centre of each map indicate the branch ostium. (Since the size and shape of the ostium varied slightly from branch to branch within each age group, the black squares indicate where a branch mouth was present in any image.) Spot number is expressed per grid square; spot area is expressed per spot. Spot, non-spot and total uptake are expressed in the same arbitrary units.

The number of hotspots and their area, and the amount of EBD fluorescence in hotspots, outside hotspots and in both compartments combined, averaged for each grid square, are mapped for regions of aortic wall around intercostal branch ostia in immature and mature rabbits in Fig. 1. (Note that every maps in this and subsequent figures use a color bar that ranges from the lowest to the highest value for that map.) Within each age group, the patterns for all 5 metrics were broadly similar, but there was a strong effect of age: in the immature group, high values for each metric tended to occur downstream of the branch ostium whereas in the mature group they occurred in four patches located at the corners of the map.

At both ages, the averaged number of spots reached a maximum of approximately one per grid square (area 56,644 μm^2^). Overall, however, mature maps had significantly more spots than immature maps (mature = 49.6 ± 5.3 spots per branch; immature = 26.2 ± 3.8 spots per branch, mean ± SEM, *p* = 0.0005, Student’s *t*-test). For each branch, the area mapped was 2.4 × 2.4 mm; we have previously shown that endothelial cells areas, measured in 600 × 600 μm regions upstream and downstream of rabbit intercostal branches, are: immature upstream, 336 ± 19 (μm^2^, mean ± SEM); immature downstream, 359 ± 8; mature upstream, 304 ± 18; and mature downstream, 453 ± 32 [33].

The peak value for the average area of hotspots within each grid square differed substantially, being around 250 pixels in immature animals but only 80 in mature ones. However, equivalent mean rather than peak values for spot areas at the two ages were 139.6 ± 8.2 pixels for immature and 125.2 ± 2.6 for mature rabbits, and were not significantly different. At both ages, uptake in those areas of the map defined as hotspots was approximately an order of magnitude lower than uptake in the larger portion of the map that fell below the hotspot intensity threshold, and non-hotspot uptake therefore accounted for the great majority of total uptake.

### 3.2 Mitosis

One immature rabbit had more than 8 times as many BrdU-positive nuclei as the average for the group, and nearly 5 times as many as the rabbit with the next highest number. It was determined to be an outlier by Chauvenet’s criterion and excluded from all further analysis.

**Fig. 2:**
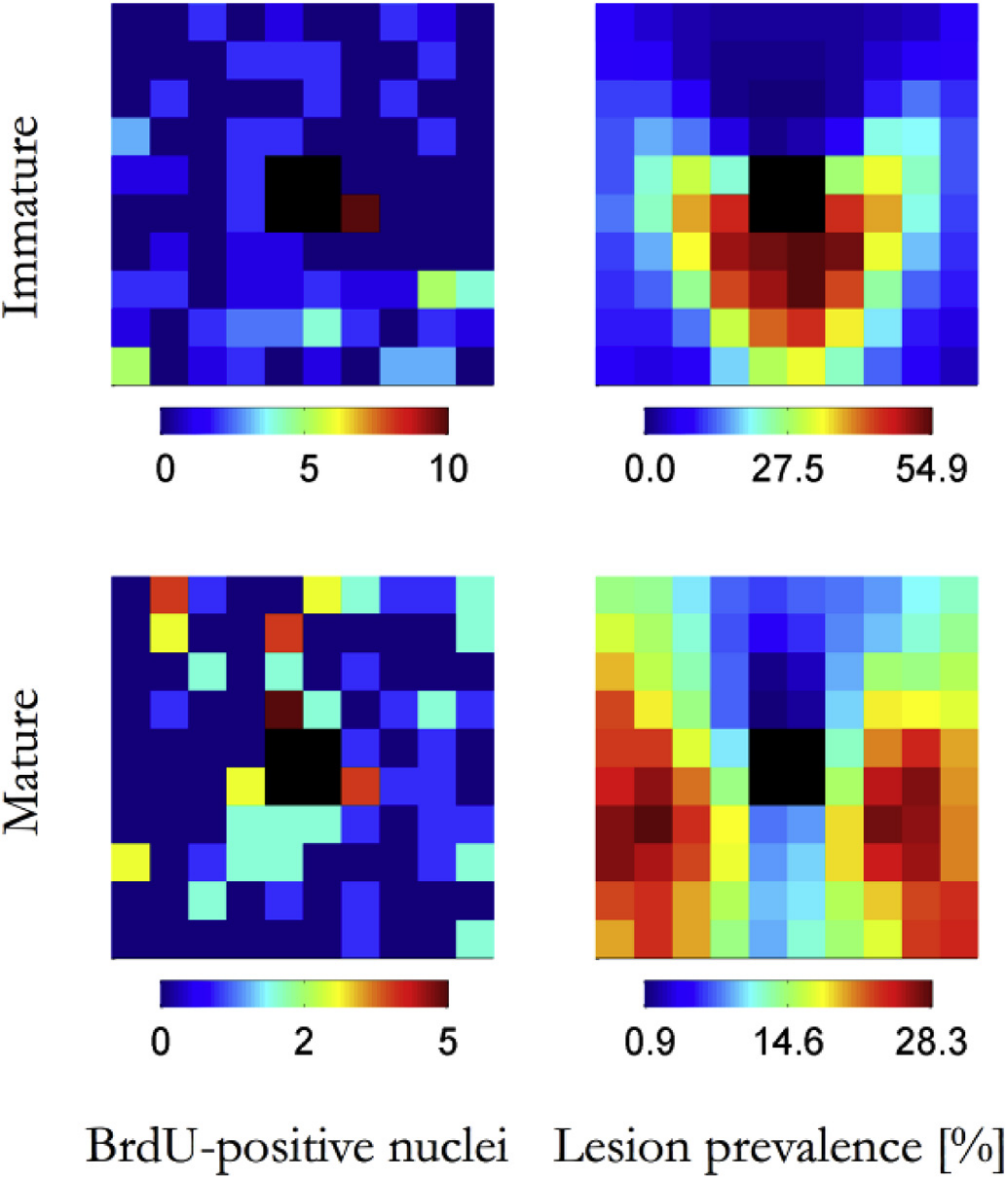
Maps of mitosis and lesion prevalence. Maps showing the average number of BrdU-positive nuclei per grid square for 16 branches from 4 immature rabbits and 20 branches from 5 mature rabbits, and maps (after [21]) showing lesion prevalence around 112 branches from 8 immature rabbits and 118 branches from 9 mature rabbits. The size and orientation of the maps are the same as in Fig. 1. The black squares at the centre of each map indicate the branch ostium.

Maps of the mean number of BrdU-positive nuclei in each grid square are shown for immature and mature animals in Fig. 2. The data were more scattered than the hotspot metrics and although the maps suggest that there were slightly more labeled nuclei downstream of the branch in immature animals, and slightly more upstream of the branch in mature ones, these differences were not statistically significant (immature, *p* = 0.15; mature, *p* = 0.14; 1-tailed *t*-test comparing the top and bottom halves of the maps).

### 3.3 Lesions

Maps of the frequency of lesion occurrence around intercostal branches in immature and mature cholesterol-fed rabbits are also shown in Fig. 2. As discussed in detail elsewhere [21], the highest frequencies are seen in an arrowhead-shaped region surrounding the downstream half of the ostium in immature branches, and at the lateral margins of branches in mature animals.

### 3.4 Spatial correlations

Median values of Pearson’s *r* and corresponding 95% confidence intervals for correlations between the different EBD measures, numbers of BrdU-positive nuclei and lesion frequencies are summarised for the immature and mature age groups in Table 1. Correlations within each age group followed the same pattern: all EBD metrics had significant positive correlations with one another and with lesion prevalence. No significant correlations were observed between any EBD measure and the number of BrdU positive nuclei. In the mature group there appeared to be a weak *negative* correlation between lesion prevalence and the number of BrdU positive nuclei, but there was no relation in the immature group. There were no significant correlations between corresponding datasets from the two age groups (e.g. mature number of hotspots vs immature number of hotspots) (data not shown).

**Table 1:** Correlations between maps.

Immature	Spot area	Total uptake	Non-spot uptake	Spot uptake	BrdU-positive nuclei	Lesion prevalence
Spot no.	0.793	0.577	0.515	0.820	0.046	0.388
	[0.678 0.870]	[0.384 0.721]	[0.306 0.676]	[0.718 0.887]	[−0.112 0.208]*NS*	[0.282 0.482]
Spot area		0.595	0.515	0.882	0.002	0.253
		[0.407 0.734]	[0.307 0.677]	[0.811 0.927]	[−0.090 0.215]*NS*	[0.091 0.478]
Total uptake			0.990	0.687	0.077	0.386
			[0.983 0.994]	[0.529 0.799]	[−0.057 0.217]*NS*	[0.261 0.490]
Non-spot uptake				0.598	0.081	0.387
				[0.411 0.737]	[−0.048 0.217]*NS*	[0.256 0.493]
Spot uptake					0.034	0.308
					[−0.093 0.180]*NS*	[0.170 0.453]
BrdU-positive nuclei						0.096
						[−0.079 0.217]*NS*
Mature	Spot area	Total uptake	Non-spot uptake	Spot uptake	BrdU-positive nuclei	Lesion prevalence
Spot no.	0.704	0.549	0.481	0.758	−0.069	0.384
	[0.545 0.814]	[0.338 0.707]	[0.254 0.658]	[0.622 0.850]	[−0.207 0.060]	[0.199 0.516]
					*NS*	
Spot area		0.545	0.454	0.830	−0.020	0.396
		[0.334 0.704]	[0.221 0.637]	[0.728 0.896]	[−0.161 0.134]	[0.233 0.522]
					*NS*	
Total uptake			0.985	0.646	−0.077	0.376
			[0.975 0.991]	[0.466 0.775]	[−0.234 0.066]	[0.160 0.537]
					*NS*	
Non-spot uptake				0.533	−0.083	0.376
				[0.319 0.696]	[−0.241 0.060]	[0.161 0.535]
					*NS*	
Spot uptake					−0.031	0.320
					[−0.161 0.110]	[0.111 0.487]
					*NS*	
BrdU-positive nuclei						−0.182
						[−0.298 −0.047]

Correlation coefficients and, in square brackets, associated confidence intervals between all pairs of the parameters measured in immature rabbits, and in mature rabbits. Correlations are not significant if the confidence intervals include zero (indicated by “NS”); however, if both confidence limits are positive, or both negative, then the correlation is significant at the 5% level.

## 4 Discussion

The main finding of the present study was a significant change with age in the pattern of hotspots of EBA uptake around intercostal branch ostia in the rabbit aorta. In immature rabbits, hotspots occurred most frequently downstream of the ostium but in mature rabbits, this region was spared - instead the hotspots occurred in two axial stripes lying either side of the branch mouth, but with sparing of the regions immediately lateral to the ostium itself. These patterns were seen in maps of the number of hotspots, their area and the amount of tracer within them; within each age group, but not between them, the three measures were highly correlated. Several previous studies have reported the immature pattern of EBA uptake, as noted above, but we believe the mature pattern is novel.

At both ages, hotspot uptake was an order of magnitude lower than non-hotspot uptake. It is possible that the proportions of hotspot and non-hotspot uptake may depend on solute size; hotspots may be more important for the uptake of LDL. An interesting finding was that the map of hotspot uptake at each age correlated with the map of non-hotspot uptake (and hence, of course, with the map of total uptake). Indeed, within each group the three maps were visually indistinguishable. At minimum, this correlation suggests that there may be common underlying mechanisms, perhaps related to mechanical forces [34], [35], which determine variation in both hotspot and non-hotspot uptake. The data are also consistent with the speculation that there is not a binary distinction between the two types of transport, but instead a continuum; for example, non-hotspot and hotspot uptake could both reflect transport through inter-endothelial cell junctions, with the width of the junction and hence the local rate of transport varying in a continuous fashion. A distribution of junctional widths, varying between grid squares, and an arbitrary width at which transport was termed hotspot rather than non-hotspot would explain the spatial correlation between the number of hotspots, their area and hotspot uptake. (Larger junctions would experience much higher convective velocities, which depend on the 4th power of cylindrical pore radius, leading to faster advection of tracer and, presumably, to greater spreading of the spot.)

**Fig. 3:**
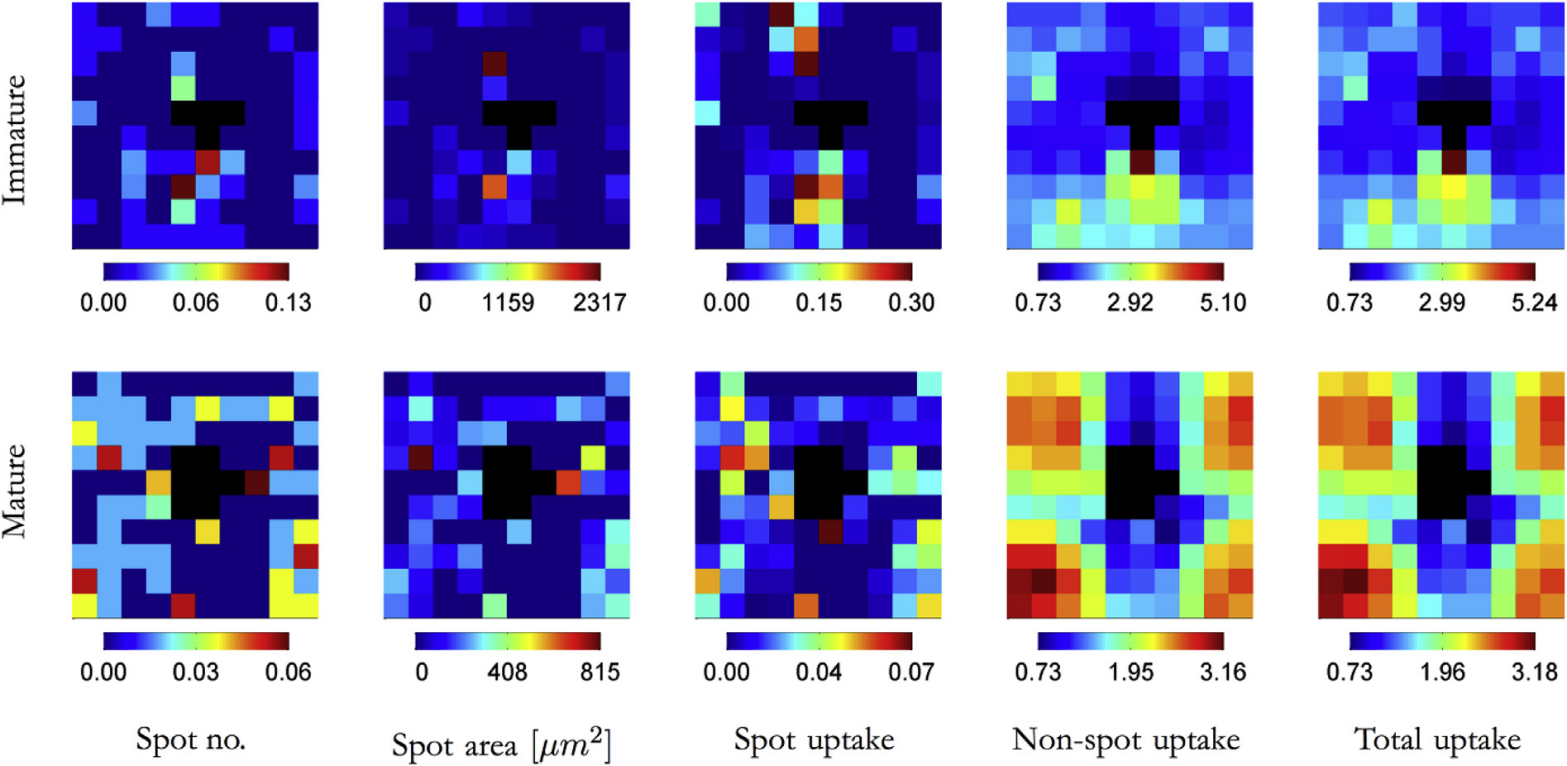
Maps for larger hotspots. Average EBA uptake maps, as in Fig. 1 except that spots were filtered so that only those larger than 450 pixels (4552 μm^2^) rather than 50 pixels (506 μm^2^) were selected. Presentation is the same as in Fig. 1.

An alternative explanation is that we included smaller hotspots than those identified in previous studies, and that we therefore counted what previously might have been considered non-hotspot uptake in our hotspot uptake, confounding the two mechanisms. For the results described so far, we used an area threshold of 50 pixels (506 μm^2^), approximately equivalent to one endothelial cell, in our definition of a hotspot. To test whether this was too small, we also processed images using a nine-fold larger area threshold of 450 pixels (4552 μm^2^). That is equivalent to a circular spot with one half of the average radius of 75 μm reported by Shou et al. [36] for 1-min HRP uptake. (The same time and tracer were used by Stemerman et al. [10]). Although the mean maps grew sparser with the increased filter size, the general patterns of hotspot occurrence remained the same (compare Fig. 3 with Fig. 1), consistent with the concept of a continuum of junctional widths.

Since many previous authors have noted that hotspots occur where endothelial cells are undergoing mitosis, we investigated whether patterns of mitosis change with age in the same way that hotspots do. We used a different strategy from those previously employed; we investigated mitosis and hotspots in different animals and then statistically compared the resulting maps. This has the advantage that a cumulative record of mitosis over several days can be obtained by administering BrdU in drinking water, increasing the number of mitotic cells detected; the use of separate animals avoids the possibility that this potentially mutagenic agent would itself modify transport. Identification of BrdU by immunofluorescence is likely to give a lower error rate than identifying dividing cells by visual assessment of haematoxylin stained nuclei, the method employed in much previous work. BrdU is incorporated into all newly synthesised DNA but we estimate that DNA repair will lead to a nucleus having only 1/3000^th^ of the BrdU incorporation resulting from mitosis. Its incorporation identifies cells in S phase but so long as the large majority of these cells go on to M phase (when intercellular junctions are disrupted), then our maps of S phase are equivalent to maps of mitosis.

No significant correlation was found between mitotic cell prevalence and EBA patterns in either age group. Indeed, unlike the result obtained for the distribution of hotspots, there was no significant difference between the mitosis patterns at the two ages. The lack of correlation between EBA measures and mitosis indicates that mitosis is not the most significant cause of leaky junctions. This does not necessarily disagree with the observation by Lin et al. [12] that 99% of the mitotic cells occur within EBA hotspots, since only 30% of the hotspots they identified had mitotic cells present within them. Because our method of hotspot segmentation is more sensitive than the manual identification used in earlier studies, it is likely that an even smaller percentage of hotspots in our data had mitotic cells occurring within them. Indeed, the average number of mitotic cells per branch was 20.6% of the average number of spots per branch in the immature group and 7.5% in the mature group, despite the 48-h window for detecting mitosis and the 10-min window for hotspots. (The averages were 6.2 mitotic events per immature branch and 3.9 per mature branch.) The lack of a strong relation between mitosis and hotspots is consistent with the view that, on the whole, non-hotspot and hotspot uptake represent an arbitrary division of a continuum rather than two distinct processes, although of course mitotic (and apoptotic and necrotic) endothelial cells may make a minor contribution that is of a qualitatively different type.

If mitotic events are not the main cause of the change with age in EBA uptake, then other factors need to be considered. Our early studies of the uptake of rhodamine-albumin showed that the mature pattern is dependent on endogenous NO synthesis, being abolished by the eNOS inhibitor *N*^*G*^-monomethyl-l-arginine, but the immature one is not [37], [38]. That suggests an age-related change in the signaling events controlling patterns of uptake. However, the transport data were obtained only along the centerline through the branch; a more recent study, employing en face mapping of transport, suggested that disruption of the NO pathway is associated with only quite subtle changes to transport in the mature animals [30]. Another possibility is that mechanical forces change with age. In rabbits there is a change in the helicity of flow in the descending aorta, reflecting an alteration of aortic taper, and a consequent change in the pattern of wall shear stress around branch ostia, particularly in the degree of how multidirectional the shear stress is during the cardiac cycle [39], [40], [41].

Finally, we consider the relation between EBA uptake and lesion frequency. Our data show significant positive correlations at both ages between maps of lesion frequency and all hotspot measures, non-hotspot uptake and total uptake. Lesion frequency, like these measures, was greater downstream of branches in immature rabbits and in axial streaks either side of the ostium in mature rabbits. However, inspection of the maps does reveal discrepancies between the transport and lesion patterns within each age group. In particular, lesion prevalence at both ages is high lateral to the ostium, where EBD uptake is low; the discrepancy is particularly striking in the mature age group but, once identified, can be discerned in the immature group too. Our previous studies of rhodamine-albumin uptake [28], [30] give a pattern that more closely resembles the lesion frequency maps; uptake is high lateral to the branch mouth. Hence there appears to be an issue with the uptake of EBA.

EBD inhibits endothelium-dependent relaxation [42] and it might therefore alter the mature pattern of transport, since that is NO-dependent to some extent (see above). In previous studies, long-term (3 h) aortic uptake of EBA, assessed along the centerline of the renal artery branch, was greater downstream than upstream of the ostium in immature rabbits, as expected, but this pattern was not abolished with increasing age; however, when EBA circulated for shorter times (15–20 min), the downstream pattern was abolished or reversed in rabbits approaching maturity, as seen with rhodamine-albumin [43], [44]. This suggests that long-term exposure to EBA may influence transport but short-term exposure does not. Exposure to EBA was restricted to 10 min in the present study to minimize modification of transport patterns by the EBA itself.

**Fig. 4:**
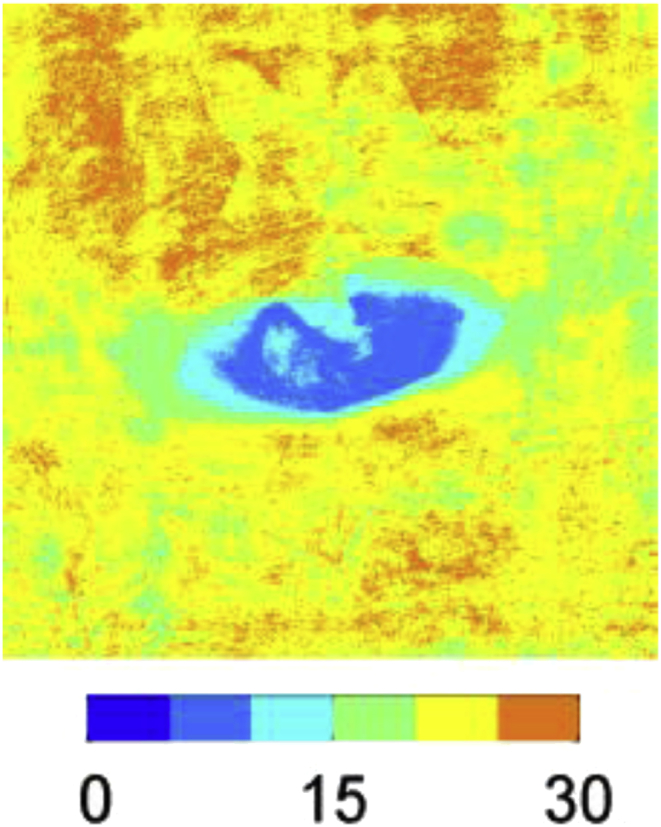
Map of autofluorescence. Levels of autofluorescence surrounding branch ostia, indicating the distribution of fibrous proteins. The image, which shows an average of maximum intensity projections for 6 branches, has the same size and orientation as the maps in Fig. 1. Intensity in arbitrary units.

A second possibility is that since EBD binds preferentially to elastin and collagen once the tracer enters the wall, its observed distribution may depend not only on transport rates but also on the amount and location of these proteins. Elastin and collagen are the primary source of tissue autofluorescence, so we investigated this possibility by scanning 6 intercostal branch ostia from a mature rabbit not administered EBA, using similar methods to those described above except that excitation and emission wavelengths were reduced to 458 nm and 490–530 nm, respectively; there is negligible autofluorescence at the longer wavelengths used to image EBA. The average map (Fig. 4) revealed regions lateral to the branch mouth with lower autofluorescence intensity than upstream and downstream regions, consistent with the view that the discrepancy between EBA and rhodamine-albumin uptake is caused by lower concentrations of elastin and/or collagen in these areas.

A number of potential limitations of the present study need to be considered. First, the size of the animals combined with the plasma concentration of tracer required for fluorescent detection made it impracticable to examine hotspots of LDL uptake. (This might be feasible in mice, but they do not show the same age-related changes in lesion pattern [45]). However, a previous study [19] has examined uptake of radiolabelled LDL in immature (2–3 kg) rabbits and found that the number of hotspots was greater downstream than upstream of intercostal branch mouths, as shown here for albumin, supporting the concept of a continuum of junctional widths. Second, the rabbits each received c. 5–10 mg/kg of EBD label. The data of Lindner and Heinle [2] suggest that if this dose of EBD had been injected as free dye, up to 3% would circulate unbound. However, the permeability patterns we observed are broadly consistent with those obtained for albumin covalently labeled with rhodamine and rigorously purified of low molecular weight contaminants, for which this issue does not arise. We therefore conclude that either this level of unbound EBD does not have a significant effect on overall uptake patterns, or that premixing the EBD with albumin prior to injection leads to lower levels of circulating free label, or that the free label and albumin have similar uptake patterns. Third, the cell turnover leaky junction hypothesis of Weinbaum et al. [7] proposes that foci of high permeability arise when endothelial cells divide or die, but we only assessed division as a potential mechanism. Approximately 50% more HRP and EBA hotspots are associated with dead and dying cells than with mitotic cells [46]. Hence it is possible that a change in the pattern of dying endothelial cells might contribute significantly to the change in hotspot pattern with age. Furthermore, such behavior might be more important for LDL than for albumin [46]. Fourth, hotspots were mapped in normocholesterolaemic animals here (and in the previous studies that we have cited), but inferences are drawn about their relation to lesion patterns seen in hypercholesterolaemic animals. Nevertheless, the rare spontaneous lipid deposition that occurs in normocholesterolaemic rabbits shows similar age related changes in distribution around intercostal ostia [22], suggesting that hypercholesterolaemia accelerates the process but does not alter underlying mechanisms.

In conclusion, our data do show that the pattern of endothelial permeability hotspots around intercostal branch ostia changes with age and in a way that is consistent with the patterns of lesions. Hotspots were not the major route for EBA uptake and were not dominantly explained by patterns of mitosis. They may play a more significant role in the transport of larger molecules such as LDL. It is plausible that hotspots represent an arbitrary subdivision of a continuum of local permeabilities reflecting intercellular junctions of different width.

## Conflict of interest

The authors declared that they do not have anything to disclose regarding conflict of interest with respect to this manuscript.

## Financial support

This work was funded by the British Heart Foundation Centre of Research Excellence (KYC, AC), a Marie Curie postdoctoral fellowship (AC) and a BHF Programme grant (PW).
