# Supplementary material for: Role of endothelial permeability hotspots and endothelial mitosis in determining age-related patterns of macromolecule uptake by the rabbit aortic wall near branch points
Source: Atherosclerosis. 2016 Jul;250:77–83. doi: 10.1016/j.atherosclerosis.2016.05.017 (PMC4917891; doi:10.1016/j.atherosclerosis.2016.05.017)
Supplement: Supplementary file 1 [file mmc1.docx]

## On-line Supplement

**Methods**

## *Animals and diet*

Twelve immature (70±3 days, 2.10±0.09 kg; mean±SEM) and twelve mature (333±17 days, 4.00±0.16 kg) male New Zealand White rabbits (HSDIF strain, Harlan) were individually housed at 18±2 °C under a 12 h light cycle. They were fed a normal laboratory diet (9603 TRB; Harlan Teklad, Bicester UK) ad libitum.

## *Tracer uptake experiments*

EBD (Sigma) was mixed with bovine serum albumin (BSA, fatty acid adsorbed, First Link UK Ltd) in a 14:1 molar ratio in Ringers solution (composition in g/L: NaCl, 9.0; KCl, 0.25; CaCl_2_, 0.25; NaHCO_3_, 0.1). Five conscious, unrestrained rabbits in each age group were administered 100 mg/kg (2.4 mL/kg) of the resulting Evans blue-albumin (EBA) conjugate via the marginal ear vein. This step was omitted in an additional 2 animals in each age group that were used to assess levels of naturally-occurring tissue autofluorescence. After 8 minutes, all rabbits were given heparin (2000 USP units, Sigma) and, after an additional 2 minutes, an overdose of pentobarbital (Euthatal, approx. 200 mg/kg, Rhône Mérieux) by the same route.

A ventral midline incision was made, the abdominal organs were deflected and the diaphragm removed. The aorta was cannulated retrograde and flushed by perfusion with 50 mL of Ringers solution containing heparin (2000 USP unit, Sigma) from a reservoir 90 cm above the animal, and was then fixed with 10% buffered formalin (Sigma) for 30 minutes through the same cannula. The aorta from the point of cannulation to the descending arch was excised, post-fixed in 10% formalin for 24 h, transferred to phosphate buffered saline (PBS, 0.15 M, pH7.4, Sigma) and cleansed of loose adventitial tissue.

Each aorta was opened longitudinally along its ventral surface and placed luminal surface down in a coverslip-bottomed petri dish containing PBS, flattened by placing a glass disk on top of it, and examined *en face* with an inverted confocal microscope (TCS SP2, Leica Microsystems). Fluorescence from the region surrounding each intercostal branch ostium was excited at 633 nm, and emission at 660-690 nm was recorded with a x10 objective, using a 2 x 2 tile scan to increase the field of view to 3 x 3 mm (944 x 944pixels). Stacks of optical slices were acquired in the z-direction, each slice being approximately parallel to the endothelial surface. Gains were set so that a few pixels saturated in the brightest slice. A maximum intensity projection (i.e. an en face, 2-dimensional map comprising only the brightest pixel in each depthwise column of pixels) was obtained from the confocal image stack for each branch.

## *Analysis of EBA images*

To adjust maps for spatial variation in the sensitivity of the microscope, each tile had to be divided by the equivalent image of a uniformly fluorescing object. This flatfield correction was achieved by dividing each tile by the mean autofluorescence tile, obtained from the four animals not receiving tracer; variations in autofluorescence around branches are small (Clarke et al., 2012) and were reduced further by averaging tiles from different locations around the ositum. The averaging procedure is illustrated in Figure 1. The ostium was situated within one quadrant of each of the four tiles in every 2 x 2 tile scan (Figure 1A); these quadrants were masked. Since a different quadrant was affected in each tile, superimposing the 4 masked tiles then effectively resulted in the sum of three branch-free tiles. (Figures 1B, 1C). Averaging the results from all 67 branches produced the final flatfield image (Figure 1D). Figures 1E and 1F illustrate, respectively, a tile scan of EBA fluorescence before and after correction.

Since hotspots within intercostal arteries were not of interest, the ostia were masked, using manual segmentation guided by edge detection.

Hotspots in the remaining area were identified using the thresholding method of Otsu (1979), which minimises the combined intraclass variance for the above-threshold and below-threshold groups. The method can be generalised to compute several thresholds, dividing data into several levels on the basis of their intensities. Since the non-hotspot uptake (and hence fluorescence) varied from region to region, and was superimposed on the fluorescence from the hotspots themselves, the generalised method was applied locally, in a sampling window, to remove variation, and then globally. The use of four levels in both local and global thresholding and the size of sampling window were chosen on the basis of convergence testing (see **Validation**, below).

The detailed implementation was as follows. A 100 x 100 pixel (318 x 318 μm) sampling window was placed in the top left hand corner of the whole image, and pixels within it were placed in one of four intensity categories by computing Otsu thresholds for the sub-image. This gave each pixel a value from 0 - 3; these values were placed in the corresponding pixels of a new image. The sample window was then moved one pixel to the right and Otsu thresholds were recalculated. The new Otsu values were again placed in the new image, being added to the one obtained in the previous sub-image for each pixel that had been examined twice. This process was repeated, the sample window moving in a raster, until the whole of the original image had been covered. Each pixel in the new image could have had a value between 0 and 3 times the number of times it had been included in a sample window. A 100-pixel border was cropped from the new image to ensure that all pixels had been included in the same number of local thresholding calculations. To determine which of these locally-thresholded values should indicate the presence of a hotspot, three Otsu thresholds were computed for the entire new image, placing pixels into four categories; pixels in the top category were considered to be part of a hotspot.

Individual hotspots were identified using 8-connectivity in the resulting binary image and were filtered by area to reduce noise: apparent hotspots smaller than 50 pixels (506 μm^2^), which is similar to the average surface area of endothelial cells in the vicinity of a rabbit intercostal ostium (Nerem et al., 1981), were excluded. A filter of 450 pixels (4552 μm^2^) was also applied in some cases to identify a subpopulation of larger spots. The number, intensity and area of hotspots were computed from the filtered binary image. Experiments with a series of test images demonstrated the ability of the method to correctly identify the original number of spots and their areas despite the use of spots of varying size and shape and the addition of high and low frequency noise (see **Validation**, below).

The filtered binary image was used not only to identify hotspots but also to measure non-hotspot transport: it was applied as a mask to the flatfield-corrected image to obtain an image of the residual albumin uptake.

## *Cell division experiments*

Five rabbits in each age group had their drinking water supplemented with 1.6 mg/mL 5-bromo-2-deoxiuridine (BrdU, Sigma) for 48 h. The solution was freshly made twice a day. At the end of this period, the animals were killed and their aortas flushed, fixed in situ and excised as described above. Aortas were transferred to PBS and cleaned of loose adventitial tissue without post-fixation. For antigen retrieval, they were then immersed in 0.01 M citrate buffer (pH 6.0) for 1 h, opened ventrally, clamped between 2 microscope slides and maintained at 100 °C for 15 minutes.

The tissue was washed in PBS and blocked with normal goat serum (1:100 dilution, Amersham Biosciences, UK) for 30 minutes. It was then incubated for 1 h with a mouse anti-BrdU monoclonal antibody (Becton Dickinson; 1:20 dilution with 1% BSA in PBS), washed with 1% BSA in PBS, and incubated for 1 h with fluorescein-conjugated goat anti-mouse IgG (KPL, Gaithersburg, USA; 1:20 dilution in Tris-buffered saline, pH7.6, Sigma). All incubations were conducted at room temperature. Nuclei were counterstained with 0.5 mg/mL propidium iodide (PI; Eugene, Netherlands) for 1 minute and the tissue studied immediately.

Aortas were mounted for confocal microscopy as described above. Fluorescein-labelled antibodies were excited at 488 nm and PI-stained nuclei at 543 nm. Emission was recorded with a x63 glycerol immersion objective at 510-530 nm and 600-640 nm, respectively. The 2nd and 4th pairs of intercostal branch ostia were examined in all 10 animals. A 12 x 12 tile scan, centered on each ostium, was used to obtain a field of view of 2.86 x 2.86 mm, as close in size as practicable to that obtained in the EBA experiments with a different objective. The 144 stacks of optical slices were examined for fluorescein and PI fluorescence, and the number and location of fluorescein-stained nuclei in the endothelial layer of each 238 x 238 μm tile (462 x 462 pixels) was recorded.

## *Spatial correlation statistics*

To enable comparison of images of differing resolution and size, datasets were cropped and re-sampled to obtain 10 x 10 grids covering a 2.4 x 2.4 mm region of interest (ROI) centered on the ostium. To obtain lesion prevalence maps, the same procedures were conducted for the maps obtained in our previous work (Cremers et al., 2011), in which 8 immature (64 days, 1.79±0.01 kg) and 9 mature (183 days, 3.63±0.07 kg) male New Zealand White rabbits of the HSDIF strain were fed a normal diet supplemented for 8 weeks with 1% cholesterol. Pair-wise rank correlation (Pearson's *r*) was used to determine the spatial correlation between pairs of hotspot metric maps and 95% confidence intervals (CIs) were calculated using the Fisher transformed z' statistic. Paired correlation was not possible for comparisons between hotspot, mitosis and lesion maps, so an *r*-statistic distribution was estimated, using a bootstrapping method to calculate 95% CIs at both individual and average map level (Rowland et al., 2015). When this method is applied to unpaired data, it gives more conservative estimates of the CIs than would have been calculated if a paired test had been possible.

**References**

Clarke LA, Zahra Mohri, Weinberg PD. High throughput en face mapping of arterial permeability using tile scanning confocal microscopy. Atherosclerosis. 2012;224:417-425.

Otsu N. A Threshold Selection Method from Gray-Level Histograms. IEEE Trans. Syst. Man Cybern, 1979;9:62-66.

Nerem RM, Levesque MJ, Cornhill JF. Vascular endothelial morphology as an indicator of the pattern of blood flow. J Biomech Eng. 1981;103:172-176.

Rowland EM, Mohamied Y, Chooi KY, Bailey EL, Weinberg PD. Comparison of statistical methods for assessing spatial correlations between maps of different arterial properties. J Biomech Eng. 2015. doi: 10.1115/1.4031119. [Epub ahead of print]


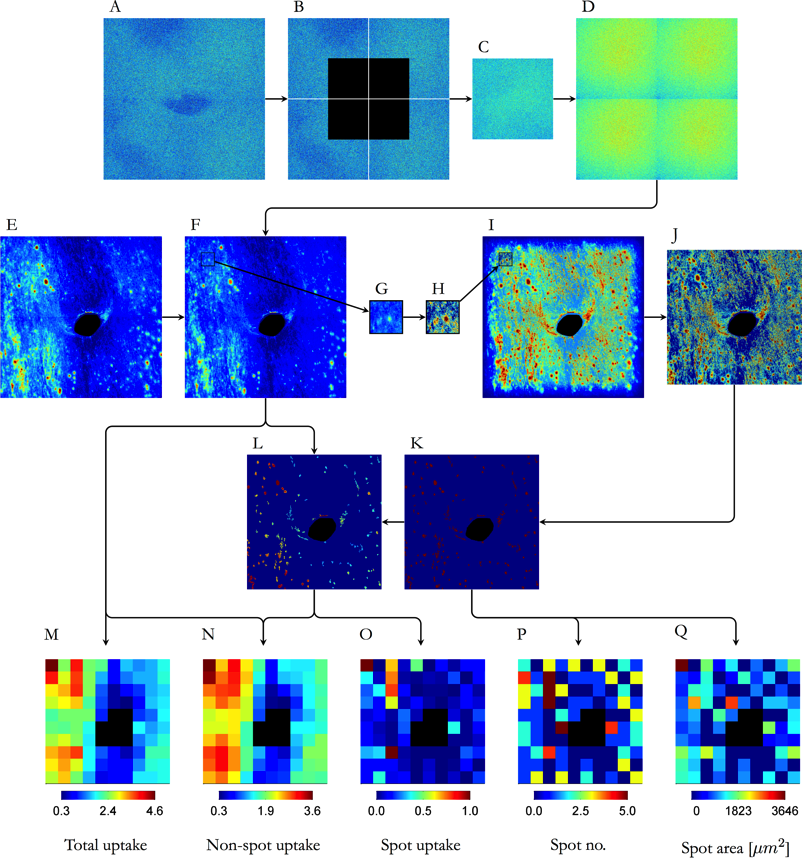


Figure 1. Image processing steps involved in hotspot segmentation

The workflow is illustrated using typical en face images from mature animals, with mean aortic flow from top to bottom. **A.** Maximum intensity projection of a 2 x 2 tile scan covering a 3 x 3 mm area of aortic wall centered on an intercostal branch ostium, showing autofluorescence in an animal not administered tracer. **B.** Superposition of mask to block the ostium, and separation of constituent tiles. **C.** Average of all four quadrants from (B). **D.** 2 x 2 tile flatfield image reconstructed from 4 images of the type shown in (C) but using data from all 67 autofluorescence branches. **E.** Typical image from a mature animal administered tracer. **F.** The corresponding flatfield-corrected image (inset:100 x 100 pixel sampling window). **G.** 100 x 100 pixel image cropped from F. **H.** 4-level Otsu thresholding applied to (G). **I.** Reconstruction of all 100 x 100 pixel images after 4-level Otsu thresholding. **J.** The same image after removal of a 100-pixel-wide border, to eliminate the edge artifact introduced during local thresholding. Total uptake (**M**) is quantified using this image. **K.** A binary image showing spot locations, used to generate spot counts (**P)** and to measure spot areas (**Q**). **L.** The top level of pixels in (J), which are interpreted as hotspots, coloured by their original intensities in (F) and used to quantify spot uptake (**O**). **N** is obtained by subtracting (L) from (F) and then calculating uptake as in image (M).

## Validation

## *Validation of the number of Otsu levels for identifying hotspots*

The use of four Otsu levels was based on the results of a convergence test that determined the effect of the number of levels on the total number and area of hotspots in a subset of the images. Figure 2 details the outcomes of using 2 to 5 Otsu levels. In each case, the number of levels used for local thresholding was also applied in the global thresholding. That paring was deemed optimal from inspection of the image histogram after the local threshold had been applied: when three local levels were applied, for example, the resulting histogram had three distinct populations of pixel intensities, so three global levels achieved the best separation of hotspots.

For low numbers of Otsu levels (eg: two), the method was inherently unstable due to sampling effects. Increasing the number of Otsu levels attenuated this effect and convergence was subsequently achieved. The use of four levels in both local and global thresholding identified a substantially smaller number of hotspots than the use of fewer levels, but using more levels did not change the result. Thus we concluded that using 4 Otsu levels at each thresholding stage was optimal. Effects of local image size were also investigated systematically; there was a strong influence of size on segmentation for sizes up to 100 pixels, so this value was chosen for the main study.

| 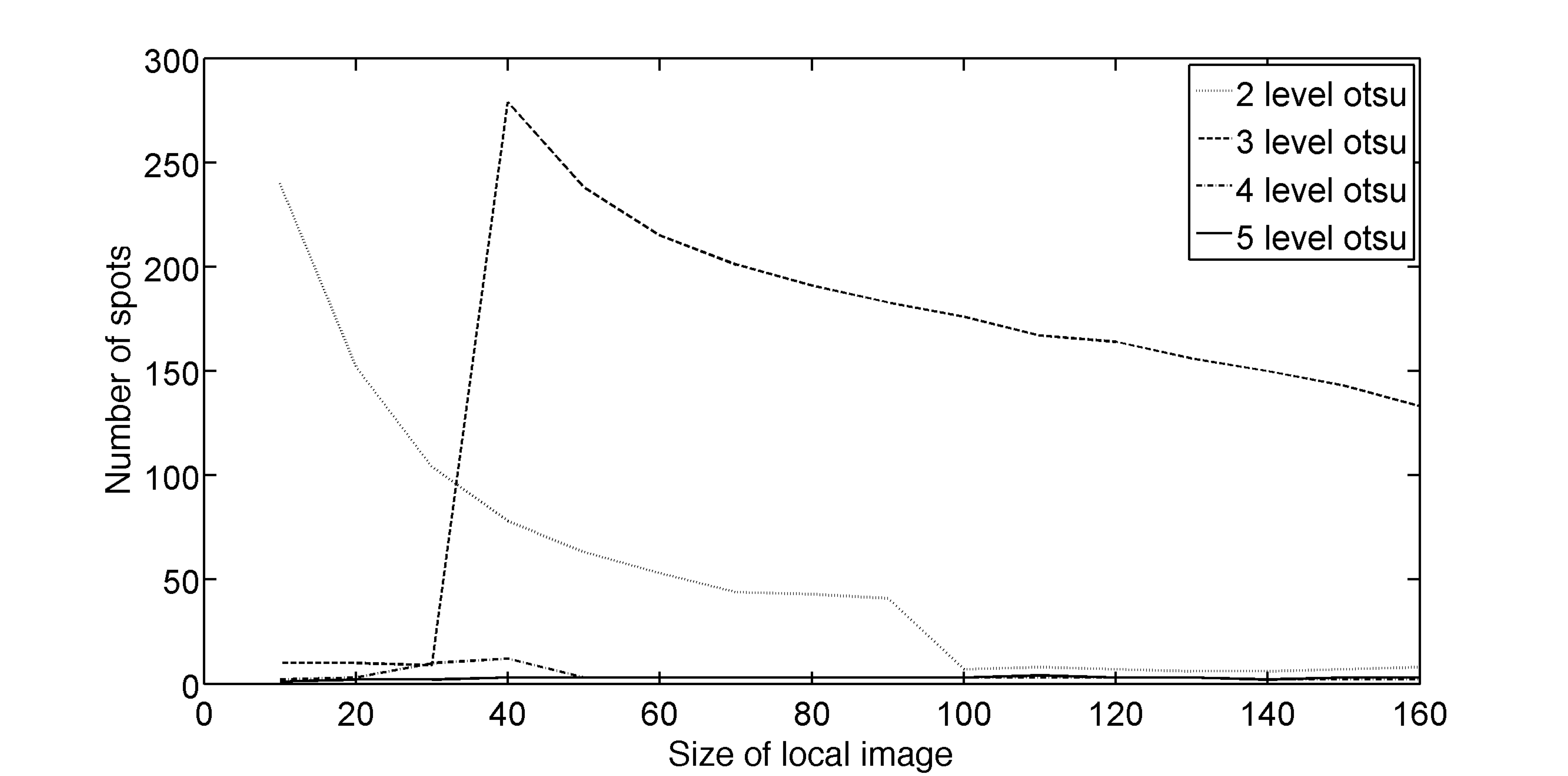 |
| --- |
| 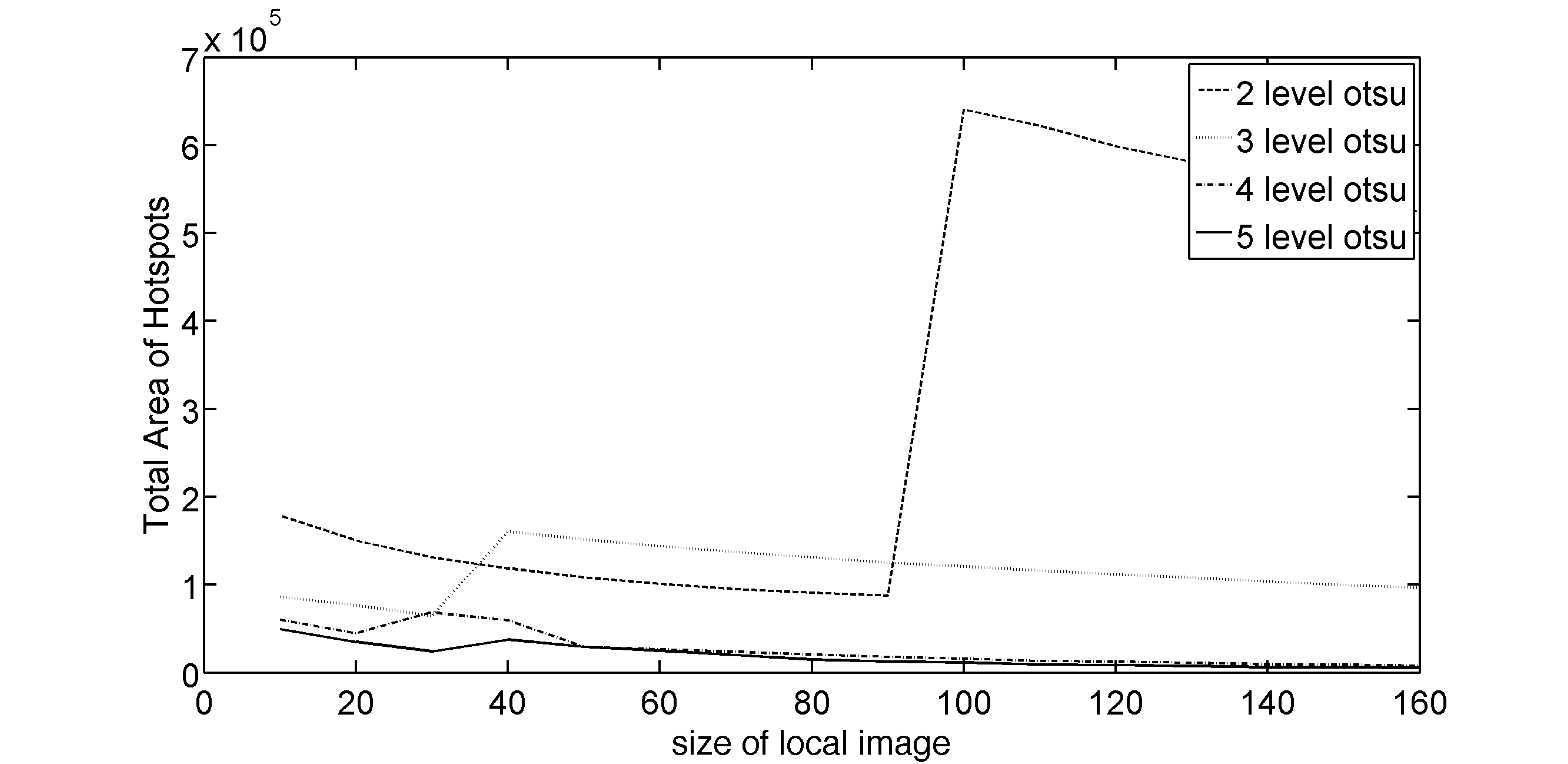 |
| Figure 2. Optimisation of segmentation  Determining the optimum number of Otsu levels at both local and global thresholding stages: the total number and area of spots converged when 4-level Otsu thresholds were applied to sub-images >100 pixels in size. |

## *Validation of the segmentation algorithm on test images*

A series of test images were used to assess the method’s accuracy in determining the number and area of hotspots. The test images contained a known number of spots super-imposed on a background with low frequency noise, simulating the more smoothly varying, non-hotspot uptake of EBA. A second test image was generated from the first by adding high frequency noise, to simulate noise introduced during imaging. Different spot sizes and shapes were also used. In all tests, the method correctly identified the original number of spots and their areas - an example is shown in Figure 3.

| 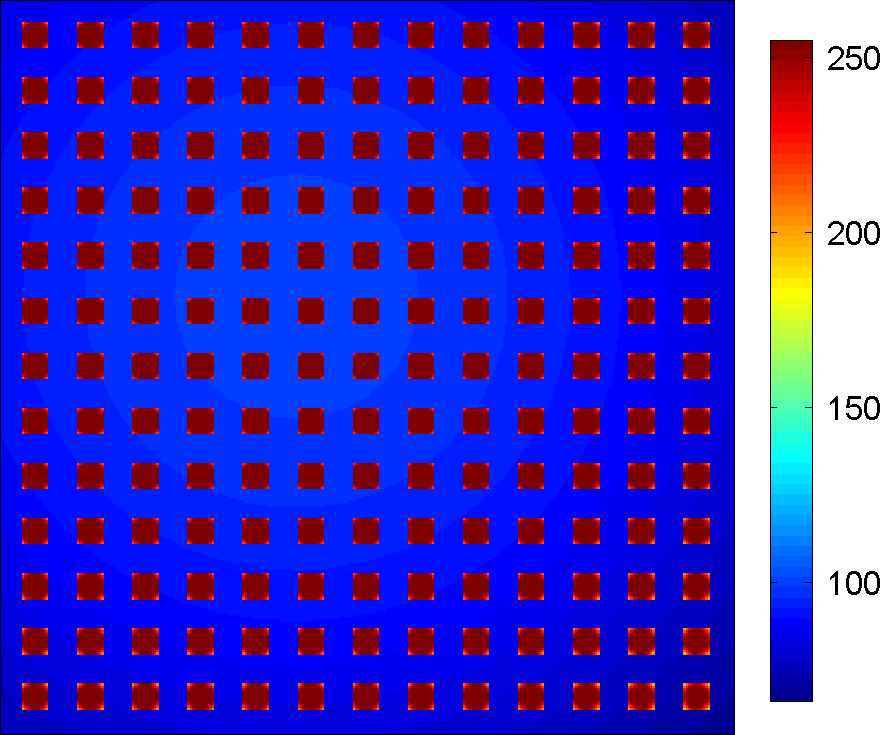 | 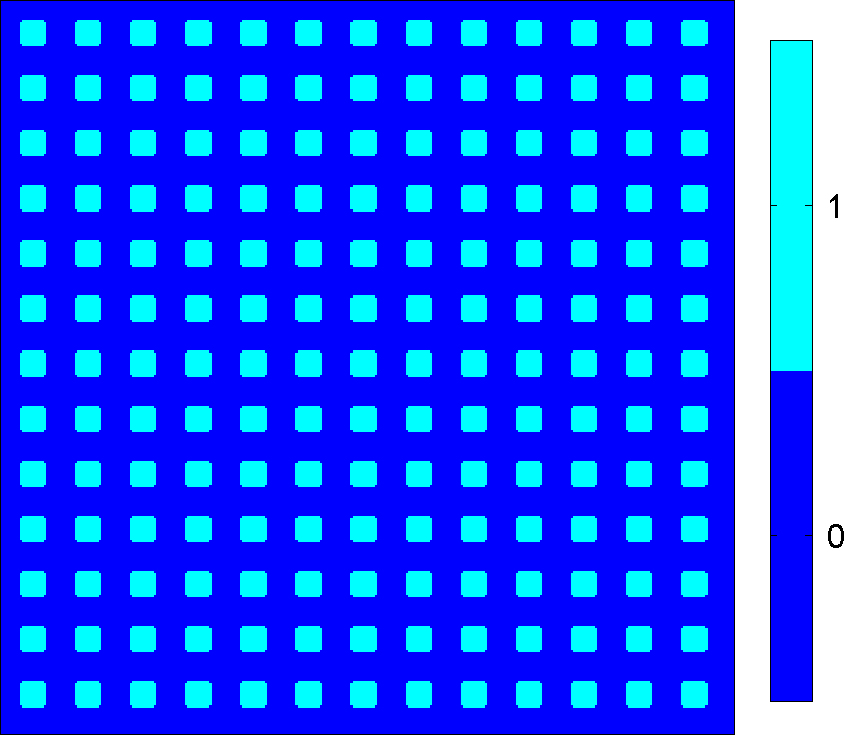 |
| --- | --- |
| (a) | (b) |
| 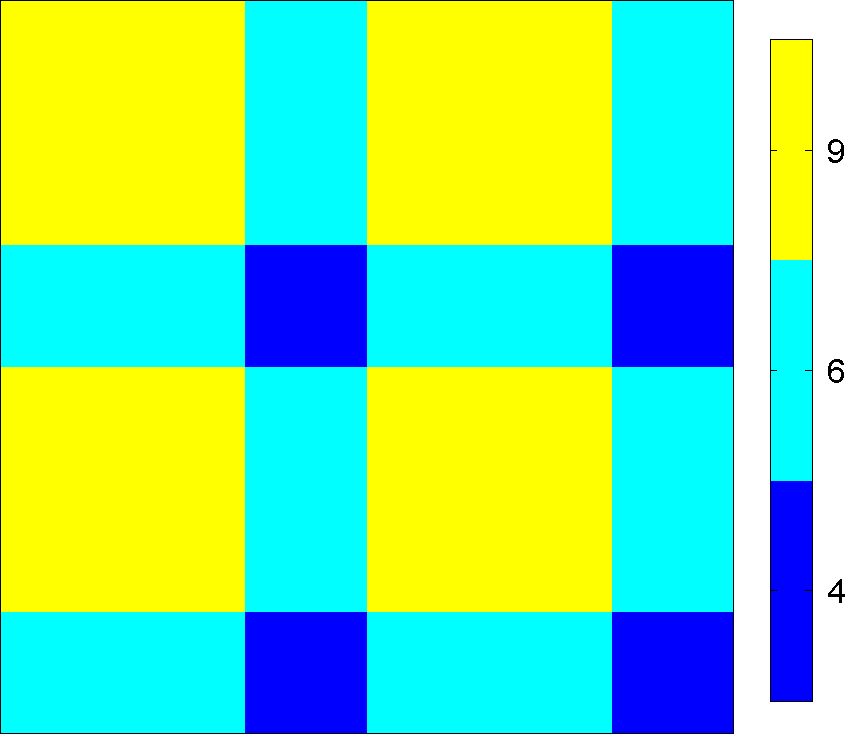 | 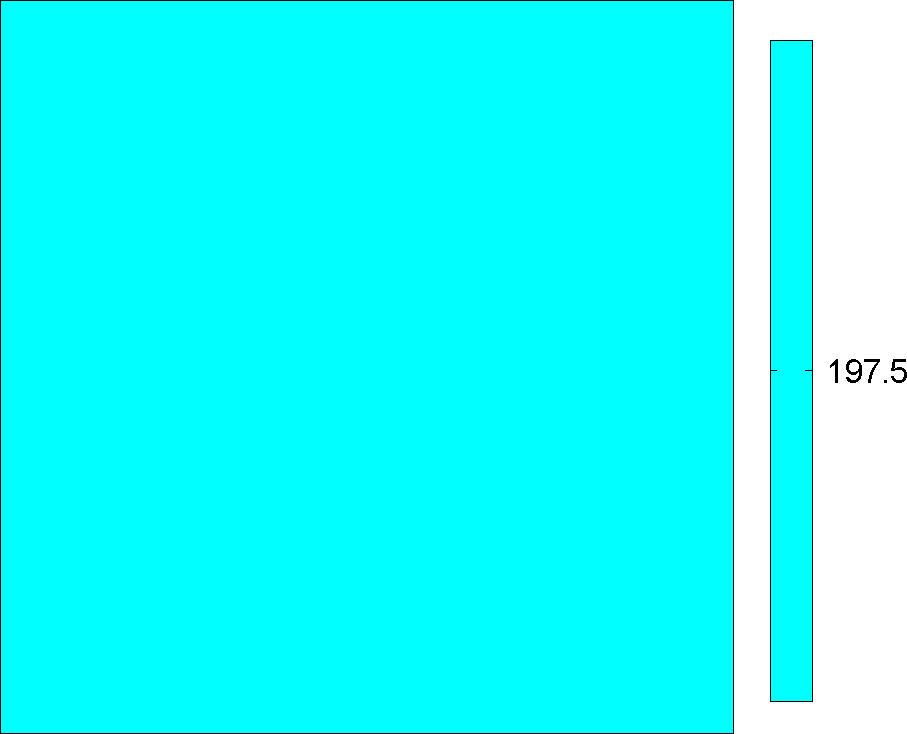 |
| (c) | (d) |
|  | |

Figure 3. Validation of 2-stage thresholding on test images

(a) test image containing a regular grid of square spots of equal size superimposed on a background with low frequency noise. (b) Segmented image shows complete removal of low frequency noise. (c) Number of spots within a 6x6 grid shows that the correct number of spots has been identified. (d) Average areas of spots demonstrates the accurate identification of spot boundaries
